# Quantitative ultrasound can assess the regeneration process of tissue-engineered cartilage using a complex between adherent bone marrow cells and a three-dimensional scaffold

**DOI:** 10.1186/ar1710

**Published:** 2005-03-01

**Authors:** Koji Hattori, Yoshinori Takakura, Hajime Ohgushi, Takashi Habata, Kota Uematsu, Jun Yamauchi, Kenji Yamashita, Takashi Fukuchi, Masao Sato, Ken Ikeuchi

**Affiliations:** 1 Department of Orthopaedic Surgery, Nara Medical University, Nara, Japan; 2 National Institute of Advanced Industrial Science and Technology, Amagasaki Site, Hyogo, Japan; 3 Life Science Laboratories, Life Science RD Center, Kaneka Corporation, Takasago, Hyogo, Japan; 4 Institute for Frontier Medical Sciences, Kyoto University, Kyoto, Japan

## Abstract

Articular cartilage (hyaline cartilage) defects resulting from traumatic injury or degenerative joint disease do not repair themselves spontaneously. Therefore, such defects may require novel regenerative strategies to restore biologically and biomechanically functional tissue. Recently, tissue engineering using a complex of cells and scaffold has emerged as a new approach for repairing cartilage defects and restoring cartilage function. With the advent of this new technology, accurate methods for evaluating articular cartilage have become important. In particular, *in vivo *evaluation is essential for determining the best treatment. However, without a biopsy, which causes damage, articular cartilage cannot be accurately evaluated in a clinical context. We have developed a novel system for evaluating articular cartilage, in which the acoustic properties of the cartilage are measured by introducing an ultrasonic probe during arthroscopy of the knee joint. The purpose of the current study was to determine the efficacy of this ultrasound system for evaluating tissue-engineered cartilage in an experimental model involving implantation of a cell/scaffold complex into rabbit knee joint defects. Ultrasonic echoes from the articular cartilage were converted into a wavelet map by wavelet transformation. On the wavelet map, the percentage maximum magnitude (the maximum magnitude of the measurement area of the operated knee divided by that of the intact cartilage of the opposite, nonoperated knee; %MM) was used as a quantitative index of cartilage regeneration. Using this index, the tissue-engineered cartilage was examined to elucidate the relations between ultrasonic analysis and biochemical and histological analyses. The %MM increased over the time course of the implant and all the hyaline-like cartilage samples from the histological findings had a high %MM. Correlations were observed between the %MM and the semiquantitative histologic grading scale scores from the histological findings. In the biochemical findings, the chondroitin sulfate content increased over the time course of the implant, whereas the hydroxyproline content remained constant. The chondroitin sulfate content showed a similarity to the results of the %MM values. Ultrasonic measurements were found to predict the regeneration process of the tissue-engineered cartilage as a minimally invasive method. Therefore, ultrasonic evaluation using a wavelet map can support the evaluation of tissue-engineered cartilage using cell/scaffold complexes.

## Introduction

Defects in articular cartilage (hyaline cartilage) resulting from traumatic injury or degenerative joint disease do not repair themselves spontaneously, because of the low mitotic activity of chondrocytes and the avascular nature of this type of cartilage [1,2]. Therefore, defects may require novel regenerative strategies to restore the biological and biomechanical function of the tissue. Recently, tissue engineering using cell/scaffold complexes has emerged as an approach for repairing cartilage defects and restoring cartilage function [3-5]. However, little is known about which scaffolds and which cells (chondrocytes or cells derived from bone marrow) are effective for the treatment of cartilage defects. Furthermore, the length of time required for chondrocyte maturation or stem cell differentiation into hyaline cartilage is unknown.

With the advent of new technologies in scaffold processing and cell biology, accurate methods for evaluating articular cartilage have become important. In particular, *in vivo *evaluation is essential for determining the best treatment. However, without a biopsy, which causes damage, articular cartilage cannot be accurately evaluated in a clinical context.

We therefore developed a new ultrasonic evaluation system for articular cartilage and showed that this system can quantitatively evaluate cartilage degeneration clinically [6,7]. The analysis system is based on wavelet transformation of the reflex echogram from articular cartilage. Our previous study revealed that the system could predict the histological findings for tissue-engineered cartilage [8,9]. However, it remained to be seen whether this system could accurately evaluate tissue-engineered cartilage from cell/scaffold complexes, especially the regeneration process. The purpose of the present study was to find out. Therefore, we fabricated three-dimensional scaffolds using a biodegradable polymer to engineer hyaline-cartilage-like tissue derived from adherent bone marrow cells and evaluated the tissue-engineered cartilage after implantation in rabbit cartilage defects. We investigated whether ultrasound could evaluate the regeneration process at 4 and 12 weeks after the implantation of a cell/scaffold complex. The relations between the ultrasonic examination and histological or biochemical examinations were analyzed.

## Materials and methods

### Three-dimensional PLGA scaffold

**Figure 1:**
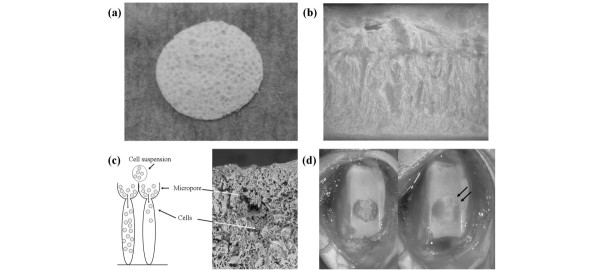
The three-dimensional poly(lactic-glycolic acid) (3D-PLGA) scaffold. The micropore side (cell seeding side) **(a) **and a cross section **(b) **of the scaffold. Schematic illustration of cell seeding (left) and scanning electron photomicrograph of cross section of cells seeded in the 3D-PLGA scaffold (right) **(c)**. The cells lie in a uniform array at the palisades, similar to hyaline cartilage. Gross appearance of a cartilage defect in the patella groove implanted with a complex between adherent bone marrow cells and 3D-PLGA scaffold **(d)**. The arrows indicate cell/PLGA scaffold.

The biodegradable scaffolds (GC Corporation, Tokyo, Japan) used in this study were described previously [10-12]. The scaffolds (5 mm in diameter, 1.5 mm thick) were composed of poly(lactic-glycolic acid) (PLGA) with a molecular mass of approximately 100,000. The outline of the scaffold construction is described below. Poly(DL-lactic-co-glycolic acid) was dissolved in dioxane added to sodium citrate particles and then frozen. The PLGA scaffold was created by a series of processes involving evaporating the solvent, washing with water to remove salts, and drying the frozen PLGA/sodium citrate. The pores at the top of the scaffold were created by the salt leaching and those at the bottom were made by the solvent evaporation. Therefore, the scaffold had micropores on the top surface and had numerous cylindrical boreholes (Fig. 1), and within the scaffold the cells lay in a uniform array at the palisade. The average pore size in the unit area on the top surface of the scaffold was 300μm. Since the micropores were present only on the top surface, the cultured cells infiltrated the scaffold after instillation of the cell suspension and did not leak out.

### Culture of adherent bone marrow cells

Twenty adult male Japanese white rabbits (3 to 4 kg) were used in this study; they were individually maintained in stainless-steel cages. The rabbits were anesthetized with a mixture of ketamine (50 mg/ml) and xylazine (20 mg/ml) at a ratio of 2:1, via a dose of 1 ml/kg injected intramuscularly into the gluteal muscle. Bone marrow was then isolated from the humeral head using an 18-gauge bone marrow needle, and 5 ml of the marrow was drawn into a 10-ml syringe containing 0.1 ml heparin (3,000 U/ml). The released cells were transferred to a T-75 flask (Costar, Cambridge, MA, USA) containing 15 ml of medium. The medium used was Eagle's minimal essential medium (MEM) containing 10% fetal bovine serum and antibiotics (penicillin, 100 U/ml; streptomycin, 0.1 mg/ml; and amphotericin B (Fungizone), 0.25 g/ml; all from Sigma Chemicals, St Louis, MO, USA). The cells were grown in a humidified atmosphere of 5% carbon dioxide at 37°C and the medium was replaced with fresh medium every 2 days. No growth factors were added. The cell culture was maintained for 2 weeks until the cells reached confluence, and then the cultured adherent bone marrow cells were released from the substratum using 0.25% trypsin and counted in a hemocytometer. The cultured cells obtained from each rabbit were reseeded onto three-dimensional PLGA scaffolds by simply dropping the cell suspension onto the scaffolds. The density of the cultured cells in a scaffold was 1 × 10^7 ^cells/cm^3^. To these composites in 35-mm tissue-culture plates we added 2 ml of fresh medium for subculture and the cultures were left to stand overnight at 37°C in 5% carbon dioxide atmosphere. During this static overnight culture, the cultured cells in the scaffold lay in uniform arrays in the palisades. The next day, the composites of adherent bone marrow cells with the three-dimensional PLGA scaffold were implanted into osteochondral defects in rabbit knee joints.

### Implantation

Under general anesthesia as described above, an anteromedial arthrotomy was performed in one knee with the joint flexed maximally. The patella was dislocated laterally and the surface of the femoropatellar groove was exposed. A full-thickness cylindrical cartilage defect (5 mm in diameter, 1.5 mm deep) was created in the patellar groove of the knee using a chisel and a disposable stainless-steel punch. After washing the knee with saline solution and drying with a swab to remove any debris, in some rabbits the defect in one knee was covered with a cell/PLGA scaffold, with the surface bearing the micropores facing the subchondral bone; this was the tissue-engineered-cartilage group (group T; *n *= 14). In a control group (group C; *n *= 6), defects were washed with saline solution and dried in the same way but were left without any further treatment. Finally, fibrin sealant (Tisseel^®^; Baxter AG, Vienna, Austria) was applied between the scaffold and the edge of the defect in group T and to the edge of the defect in group C. The wound was then closed in layers with 2-0 vicryl sutures.

The rabbits were returned to their cages and allowed to move freely without joint immobilization. The rabbits were humanely killed with an overdose of phenobarbital sodium salt at 4 and 12 weeks in group T (groups T-4 (*n *= 8) and T-12 (*n *= 6), respectively) and at 12 weeks in group C (*n *= 6). All the knee joints were opened and the cartilage surfaces were observed with the naked eye and photographed. The knee joint was dissected free from all the soft tissues and the tibia was removed. The distal femur was cut proximal to the patellofemoral joint and cartilage samples were taken. All the animals were operated on in accordance with the guidelines for animal experiments of the Nara Medical University Ethics Committee.

### Ultrasound measurements

**Figure 2:**
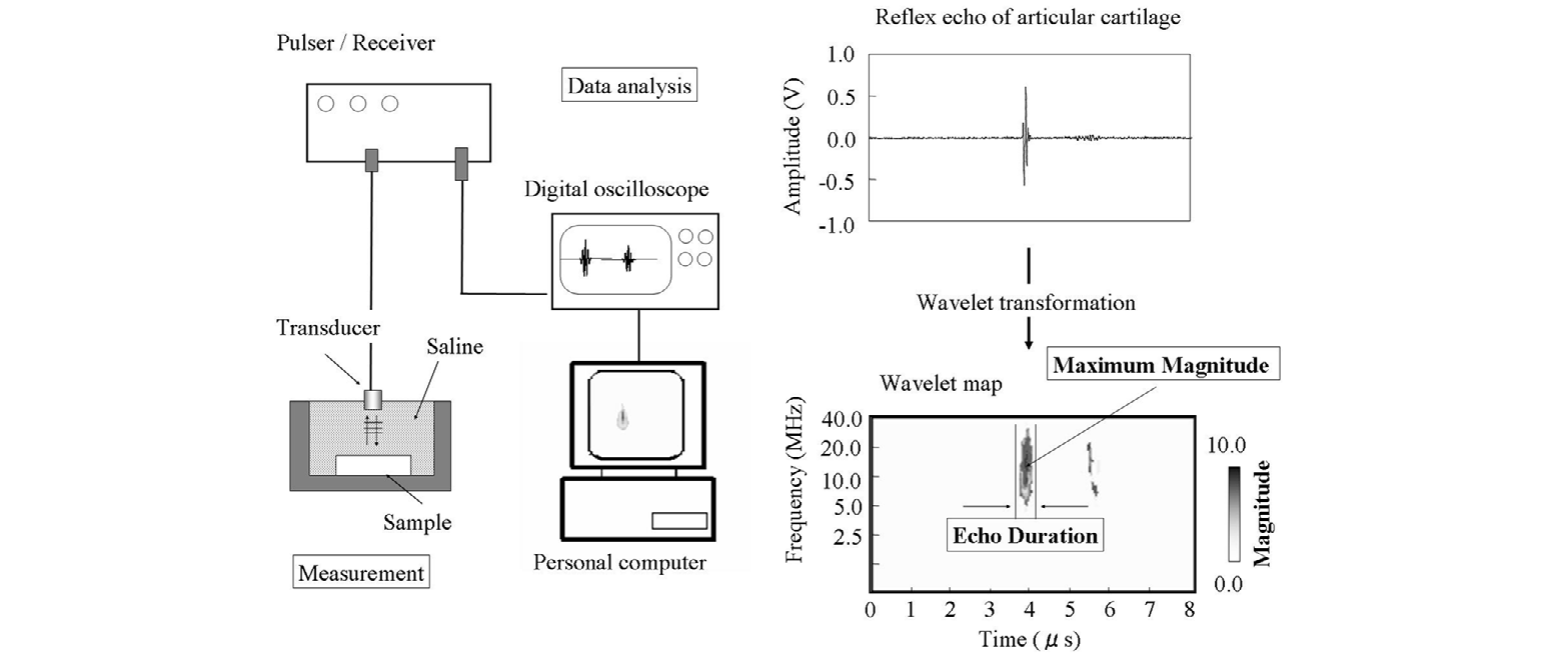
Schematic illustration of articular cartilage analysis and measurement methods of cartilage samples used in [13]. A reflex echo of articular cartilage (upper) and a wavelet map (lower) are shown on the right. The maximum magnitude is indicated by the gray scale and the percentage maximum magnitude (the maximum magnitude of the measurement area divided by that of the intact cartilage of the nonoperated knee; %MM) is used as a quantitative index of cartilage regeneration.

The ultrasonic evaluation method has been described previously (Fig. 2) [6,7,13]. Briefly, the examination was carried out in saline solution, using a transducer and pulser receiver (Panametrics Japan, Tokyo, Japan). The transducer (3 mm in diameter, 3 mm long) sent and received a flat ultrasonic wave of 10 MHz center frequency. The reflex echogram from the cartilage was transformed into a wavelet map using wavelet transformation. The wavelet transformation (W(a,b)) of the reflex echogram (f(t)) is expressed by:



where Ψ(*t*) is the mother wavelet function.

For the mother wavelet function, Gabor's function was selected. As a quantitative index of the wavelet map, the maximum magnitude was selected. This index was calculated automatically with a personal computer. The results obtained for the ultrasonic evaluation were the averages of five measurements. For the cartilage defect area, the measurement points were the center and four points at 1 mm above, below, left, and right of the center. The percentage maximum magnitude (the maximum magnitude of the measurement area of the operated knee divided by that of the intact cartilage of the opposite, nonoperated knee; %MM) was used as a quantitative index of the cartilage regeneration.

### Histological analysis

After ultrasonic evaluation, each cartilage sample was divided in two along a sagittal plane using a diamond band saw (EXAKT BS300CL; Meiwa, Tokyo, Japan). One part was used for histological analysis and the other for biochemical analysis. Histological samples were fixed in 10% formalin, decalcified in EDTA, and embedded in paraffin. Sagittal sections (5 μm thick) were prepared from the center of the defect area and stained with hematoxylin and eosin, alcian blue, and safranin-O–fast green. Sections stained with safranin-O–fast green were scored by an orthopedic surgeon under blinded conditions according to the semiquantitative histologic grading scale composed of six categories described by Caplan and colleagues [14] and were assigned a score ranging from 0 to 16 points. A high total score represented good cartilage regeneration.

### Biochemical analysis

The chondroitin 4-sulfate, chondroitin 6-sulfate, and dermatan sulfate contents were evaluated to quantify the proteoglycan content using high-performance liquid chromatography analysis [15]. The hydroxyproline content was evaluated to quantify the collagen content [16].

### Statistic analysis

All data in this study are reported as means ± standard deviations. Differences were analyzed using the nonparametric Mann–Whitney *U *test. Pearson correlations were performed to determine the associations between the ultrasonic data and the histological data. The significance level was set at *P *< 0.05.

## Results

### Ultrasonic analysis

**Figure 3:**
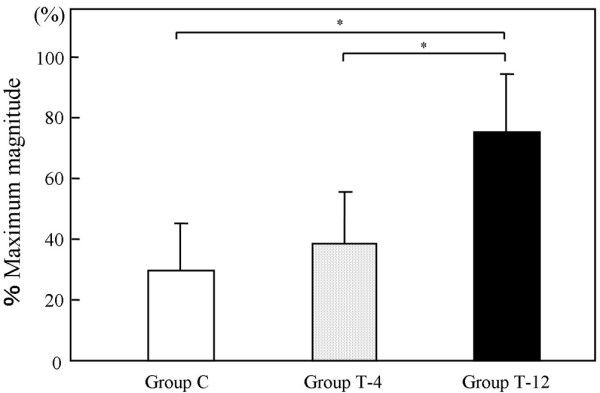
Bar graph representing ultrasonic findings in rabbits with cartilage defects treated with cell/scaffold implants. Group C, control defect group; Group T-4, tissue-engineered-cartilage group at 4 weeks after implantation; Group T-12, tissue-engineered-cartilage group at 12 weeks after implantation. The error bars represent the standard deviation of each group. The percentage maximum magnitude is expressed as the maximum magnitude of the measurement area in the operated knee, divided by that of the intact cartilage of the opposite, nonoperated knee. *P < 0.05 on the nonparametric Mann–Whitney *U *test.

The %MM values were 29.8 ± 15.1% in group C, 38.8 ± 16.9% in group T-4, and 76.5 ± 18.7% in group T-12 (Fig. 3). Significant differences in the %MM were seen between C and T-12 (*P *= 0.007) and between T-4 and T-12 (*P *= 0.007). The average %MM in group T-4 was higher than that in group C, but the difference was not significant (*P *= 0.32).

### Histological findings

**Figure 4:**
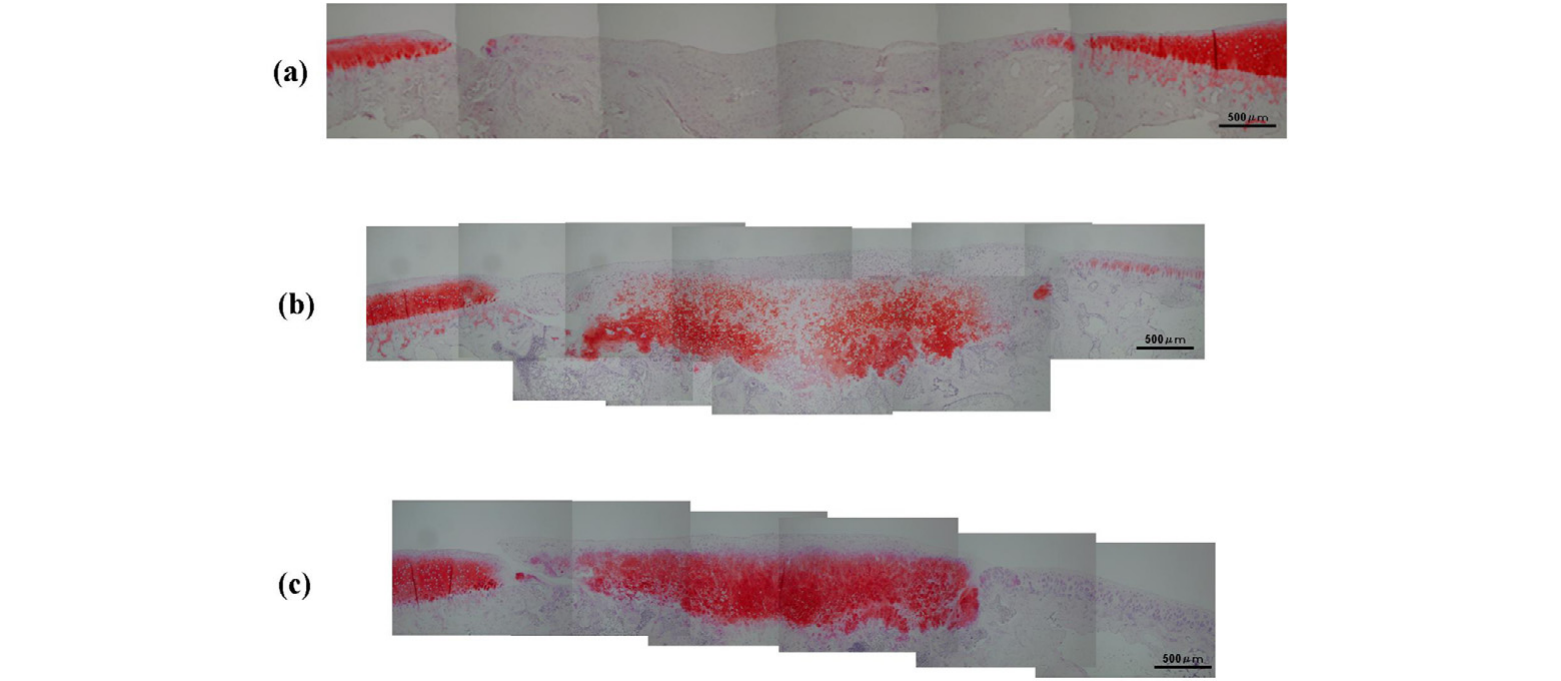
Photomicrographs of cartilage defect lesions in rabbits. **(a) **Group with control (untreated) defects (group C); and groups given tissue-engineered cartilage implants at **(b) **4 weeks after implantation (group T-4) and **(c) **12 weeks after implantation (group T-12). Safranin-O–fast-green staining; original magnification × 2.5.

**Figure 5:**
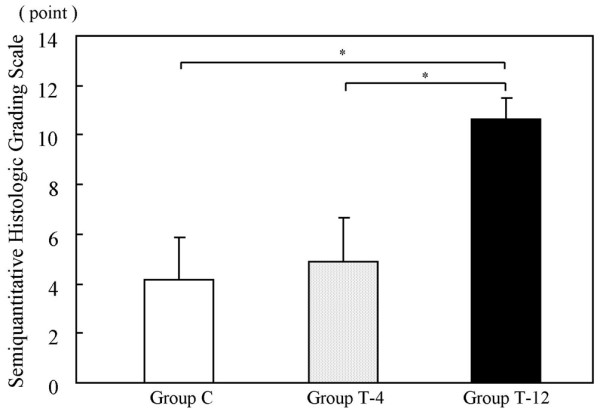
Bar graph representing semiquantitative histologic gradings for the three groups of rabbits with cartilage defects. Group with control (untreated) defects (group C); and groups given tissue-engineered cartilage implants at 4 weeks after implantation (group T-4) and 12 weeks after implantation (group T-12). Error bars represent standard deviations. *P < 0.05 on the nonparametric Mann–Whitney *U *test.

**Figure 6:**
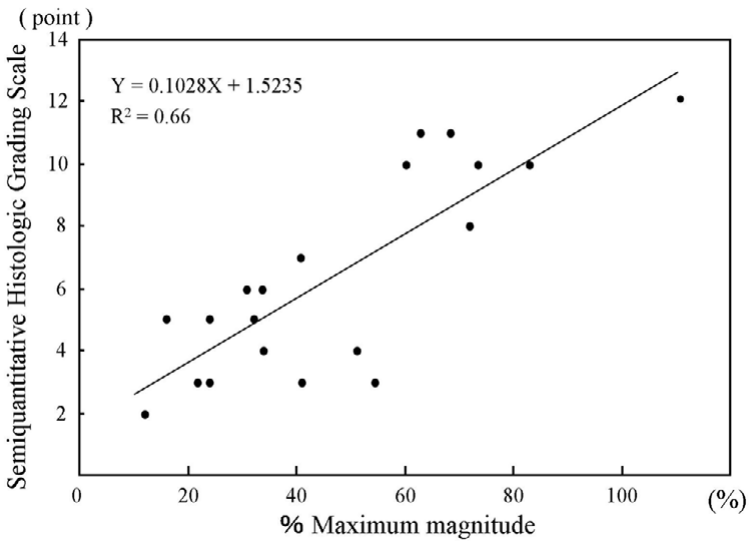
Scatter plot of ultrasound findings in rabbits with cartilage defects treated with cell/scaffold implants. The percentage maximum magnitude is expressed as the maximum magnitude of the measurement area in the operated knee, divided by that of the intact cartilage of the opposite, nonoperated knee.

In the histological findings, the defect area in group C was filled with fibrous tissue. None of the defects from group C contained any fibrocartilage or hyaline-like cartilage (Fig. 4a). The defect area in group T-4 was filled with scattered cartilage-like tissue in the scaffold. Chondroid cells with round nuclei were observed in an extracellular matrix showing normal or nearly normal safranin-O staining (Fig. 4b). The defect area in group T-12 was filled with hyaline-like cartilage, and chondroid cells lay in a uniform array in the palisades (Fig. 4c). The semiquantitative histologic grading scale scores were 4.17 ± 1.72 for group C, 4.88 ± 1.81 for group T-4, and 10.7 ± 0.82 for group T-12 (Fig. 5). Significant differences in the scores were found between C and T-12 (P = 0.004), and between T-4 and T-12 (P = 0.003). There was a correlation between the %MM from the ultrasonic examinations and the semiquantitative histologic grading scale scores for the overall results of all the measurements (*R*^2 ^= 0.66) (Fig. 6). The histological scoring showed a strong similarity to the results of the %MM values.

### Biochemical analyses

**Figure 7:**
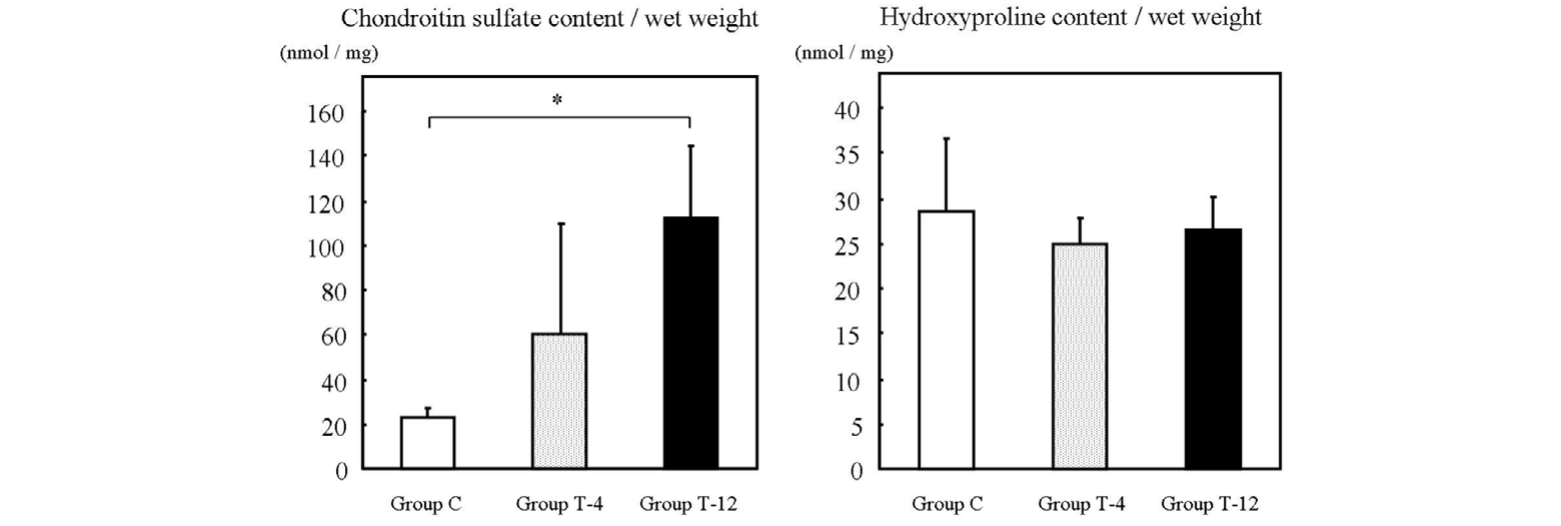
Bar graphs representing cartilage constituents in rabbits with cartilage defects given cell/scaffold implants. Group with control (untreated) defects (group C); and groups given tissue-engineered cartilage implants at 4 weeks after implantation (group T-4) and 12 weeks after implantation (group T-12). Error bars represent standard deviations. *P < 0.05 on the nonparametric Mann–Whitney *U *test.

The mean chondroitin sulfate contents were 22.9 nmol/mg in group C, 59.9 nmol/mg in group T-4, and 112.1 nmol/mg in group T-12 (Fig. 7). Significant differences in the chondroitin sulfate contents were found between group T-12 and group C (P = 0.006). The mean hydroxyproline contents were 28.5 μg/mg in group C, 25.0 μg/mg in group T-4, and 26.6 μg/mg in group T-12. There were no significant differences among the three groups. In the biochemical findings, the chondroitin sulfate content showed a similarity to the results of the %MM values.

## Discussion

In this study, ultrasonic measurements were found to predict the process of cartilage regeneration using tissue-engineered cartilage as a minimally invasive method. The main finding of the study is that the ultrasonic results were able to judge cartilage regeneration on the basis of objective data such as the %MM, since all the hyaline-like cartilage had a high %MM and the %MM increased with increasing cartilage regeneration. Therefore, ultrasound could be used to examine the microstructure of tissue-engineered cartilage using cell/scaffold complexes and investigate the length of time required for stem cells in a scaffold to differentiate into hyaline cartilage without a biopsy.

A three-dimensional porous scaffold is thought to be necessary for cartilage tissue engineering, in order to prevent the seeded cells from diffusing out of the defect site and to provide the cells with an optimal environment for cartilage differentiation [17-20]. Almost all of the scaffolds investigated have been fabricated using biodegradable polymers that have received approval for use from the US Food and Drug Administration. These polymers are favorable for the synthesis and secretion of a cartilaginous matrix, such as proteoglycans and type II collagen, and act as a physical and mechanical support for the seeded cells and their developing matrix until the polymer is remodeled by the host tissue [21]. Therefore, the clinical application of cell/scaffold complexes for cartilage regeneration is anticipated.

There are numerous clinical methods of grading regenerated cartilage at the time of surgery or arthroscopy by direct observation of the cartilage surface [22-24]. However, accurate evaluation of cartilage regeneration from cell/scaffold complexes is difficult by macroscopic observation alone. In addition, it is well established that probing cannot evaluate the cartilage condition quantitatively. As a quantitative method that could replace probing, attempts have been made to evaluate cartilage using MRI, but such *in situ *evaluation has been performed only in experimental trials [25-27]. Cartilage biopsy and histological examination have been performed to evaluate articular cartilage clinically. However, the histological score is defined by the subjectivity of the examiner, and it is still difficult to measure the degree of cartilage regeneration nondestructively. Therefore, ultrasonic evaluation using a wavelet map will be useful for supporting the evaluation of tissue-engineered cartilage using cell/scaffold complexes.

Recently, high-frequency ultrasonography was used to assess cartilage degeneration quantitatively. Chérin and colleagues [28] revealed a relation between quantitative ultrasound and maturation-related changes in rat cartilage. Jaffré and colleagues [29] reported that quantitative 55 MHz ultrasound allowed detection of early cartilage lesions due to experimental arthritis and could also detect the effects of anti-inflammatory drugs. Therefore, high-frequency ultrasonography could be useful for investigating structural changes in the cartilage matrix and evaluating the efficacy of specific therapeutic agents. However, no studies have focused on assessing tissue-engineered cartilage using high-frequency ultrasonography. In our previous work, we found that ultrasound assessment using wavelet transformation could predict the histological findings of tissue-engineered cartilage [8,9]. Using the same method, Kuroki and colleagues successfully assessed the cartilage condition of osteochondral plugs when articular cartilage defects were treated with an autologous osteochondral graft [30]. Moreover, this method has been used to assess living human cartilage under arthroscopy [7]. Therefore, ultrasound assessment using wavelet transformation should contribute to novel therapies for cartilage regeneration.

Although, the %MM was used as a quantitative index of the regenerated cartilage, what the %MM is closely related to remains unknown. Töyräs and colleagues [31] reported that ultrasound reflection could detect structural changes in the superficial collagen network and that tangential collagen fibrils act as ultrasound reflectors at the cartilage surface. Pellaumail and colleagues [32] stated that changes in high-frequency ultrasound back scatter were related to changes in the extracellular matrix collagen and most likely in its fibrillar network organization. However, these observations apparently contradict our results that the collagen content did not differ between the three groups. One explanation for this inconsistency could be differences between the reflex echoes from flat ultrasound and focal ultrasound. Another explanation could be differences in the ultrasonic frequency level (10 MHz vs 20 to 55 MHz). From an acoustic point of view, differences in the surface reflection indicate significant alterations in the acoustic impedance among regenerated cartilage samples. Therefore, the extracellular matrix, which includes not only collagen but also proteoglycans and water in the intrafibrillar space and molecular pore spaces of the extracellular matrix as hydrophilic proteoglycan aggregates, should be related to the %MM. The %MM reveals the microstructural changes in regenerated cartilage and can provide diagnostically important information about the regenerated cartilage.

Two limitations of our study should be considered. First, the maximum magnitude in our evaluation system could detect microstructural changes in a layer to a depth of 500 μm [13]. Therefore, the maximum magnitude could only evaluate the surface layer in human cartilage. However, it is of great significance to evaluate the surface layer of tissue-engineered cartilage, since this layer plays an important role in the biomechanical function of the joint. Therefore, ultrasound represents a sensitive tool for detecting regeneration of the cartilage surface in tissue engineering. Further studies using low-frequency ultrasound may provide a better assessment of the deeper layers in tissue-engineered cartilage. Second, we did not detect cartilage regeneration in living humans. However, we have previously reported relevant clinical acoustic data from human cartilage *in situ *under arthroscopy [7]. Therefore, further studies are needed to determine whether this evaluation system will prove beneficial for tissue-engineered cartilage using cell/scaffold complexes.

## Conclusion

This study reports the first results regarding the relation between quantitative ultrasound and the regeneration process of tissue-engineered cartilage. Ultrasonic evaluation using a wavelet map can support the evaluation of tissue-engineered cartilage using cell/scaffold complexes. Ultrasonic assessment using a wavelet map may contribute to the progress of tissue engineering in the musculoskeletal field, and the %MM obtained from this ultrasonic assessment can be expected to become one of the quantitative indexes of cartilage regeneration therapy.

## Abbreviations

%MM = maximum magnitude of the measurement area divided by that of the intact cartilage of the nonoperated knee; PLGA = poly(lactic-glycolic acid).

## Competing interests

The author(s) declare that they have no competing interests.

## Authors' contributions

KH conceived the study, participated in its design, and performed all the experiments. YT and HO participated in the design of the animal study. TH, KU, and JY performed the animal study. KY, TF, and MS fabricated the three-dimensional PLGA scaffold and performed the cell culture. KI participated in the design of the ultrasound analysis and performed the ultrasonic assessment. All authors read and approved the final manuscript.
